# Identification of optimal reference genes for gene expression studies in a focal cerebral ischaemia model—Spatiotemporal effects

**DOI:** 10.1111/jcmm.17284

**Published:** 2022-04-22

**Authors:** Bartosz Pomierny, Weronika Krzyzanowska, Jakub Jurczyk, Beata Strach, Alicja Skorkowska, Innesa Leonovich, Bogusława Budziszewska, Joanna Pera

**Affiliations:** ^1^ 49573 Department of Toxicological Biochemistry Faculty of Pharmacy Jagiellonian University Medical College Kraków Poland; ^2^ 49573 Department of Neurology, Faculty of Medicine Jagiellonian University Medical College Kraków Poland

**Keywords:** brain ischaemia, reference genes, RT‐PCR, transient middle cerebral artery occlusion

## Abstract

A proper reference gene (RG) is required to reliably measure mRNA levels in biological samples via quantitative reverse transcription PCR (RT‐qPCR). Various experimental paradigms require specific and stable RGs. In studies using rodent models of brain ischaemia, a variety of genes, such as β‐actin (*Actb*), hypoxanthine phosphoribosyltransferase 1 (*Hprt1*), peptidyl‐propyl isomerase A (*Ppia*) and glyceraldehyde‐3‐phosphate dehydrogenase (*Gapdh*), are used as RGs. However, most of these genes have not been validated in specific experimental settings. The aim of this study was to evaluate the time‐ and brain region‐dependent expression of RG candidates in a rat model of transient middle cerebral artery occlusion (tMCAO). The following genes were selected: *Actb*, *Hprt1*, *Ppia*, *Gapdh*, tyrosine 3‐monooxygenase/tryptophan 5‐monooxygenase activation protein, zeta (*Ywhaz*) and beta‐2 microglobulin (*B2m*). Focal cerebral ischaemia was induced by 90 min of tMCAO in male Sprague‐Dawley rats. Expression was investigated at four time points (12 and 24 h; 3 and 7 days) and in three brain areas (the frontal cortex, hippocampus and dorsal striatum) within the ischaemic brain hemisphere. The RT‐qPCR results were analysed using variance analysis and the ΔCt, GeNorm, NormFinder and BestKeeper methods. Data from these algorithms were ranked using the geometric mean of ranks of each analysis. *Ppia*, *Hprt1* and *Ywhaz* were the most stable genes across the analysed brain areas and time points. *B2m* and *Actb* exhibited the greatest fluctuations, and the results for *Gapdh* were ambiguous.

## 1 INTRODUCTION

Quantitative reverse transcription PCR (RT‐qPCR) is a widely used technique for gene expression studies. This method is highly specific, sensitive and reliable. However, each experimental setup requires proper reference genes (RGs) to reliably measure mRNA levels. The accuracy of RGs might be determined by dedicated statistical approaches: comparative ΔCt, BestKeeper, NormFinder, GeNorm or coefficient of variation analysis^1^, ^2^, ^3^, ^4^, ^5^

The aim of this study was to evaluate the time‐ and brain region‐dependent expression of RGs candidate in a rat model of transient middle cerebral artery occlusion (tMCAO), which is one of the most widely used *in vivo* model of brain ischaemia. Previous reports on RGs in brain ischaemia models were focused on the analysis of the single brain structure or single time point post‐ischaemia/reperfusion. However, considering the complex pathomechanism of brain ischaemia, cellular diversity, the spatiotemporal variability in processes induced by blood flow cessation, previous analyses provide not enough reliable data for other experimental conditions. Here, the following genes were analysed: tyrosine 3‐monooxygenase/tryptophan 5‐monooxygenase activation protein, zeta (*Ywhaz*), β‐actin (*Actb*), beta‐2‐microglobulin (*B2m*), glyceraldehyde‐3‐phosphate dehydrogenase (*Gapdh*), hypoxanthine phosphoribosyltransferase 1 (*Hprt1*) and peptidyl‐propyl isomerase A (*Ppia*). Since detrimental and repair processes that occur after ischaemia and subsequent reperfusion are dynamic the expression was investigated at four time points (12 and 24 h; 3 and 7 days) and in three brain areas: the frontal cortex (CX), hippocampus (HIP) and dorsal striatum (DS). CX and HIP in tMCAO model represent the periinfarct area, whereas DS the core of ischaemia.

## 2 METHODS

All experiments were performed on male Sprague–Dawley rats (280–320 g, Charles Rivers). tMCAO was elicited as previously described.^6^ Animals were randomly allocated into the following groups: the SHAM 12 h, tMCAO 12 h, SHAM 24 h, tMCAO 24 h, SHAM 3 d, tMCAO 3 d, SHAM 7 d and tMCAO 7 d groups. For each group, *n* = 8. Time point refers to the time lapse between the onset of reperfusion and animal decapitation and tissue collection. Using TaqMan based RT‐qPCR technique the stability of most frequently used RGs was analysed in CX, HIP and in DS (Table 1). Detailed description of the Methods is provided in the Supplemental Material. All experimental protocols were approved by the First Local Ethical Committee at Jagiellonian University and in accordance with the National Institutes of Health guidelines.

**TABLE 1:** Characteristics of studied candidates for RGs

Gene symbol	Gene name	Function	Assay ID	Assay design	Amplicon length
*Ywhaz*	Tyrosine 3‐monooxygenase/tryptophan 5‐monooxygenase activation protein, zeta	Belongs to the family of proteins mediating signal transduction by binding to phosphoserine‐containing proteins	Rn00755072_m1	Probe spans exons	104
*Ppia*	Peptidylprolyl isomerase A (cyclophilin A)	Enzyme carrying out the cis‐trans isomerization of proline imidic peptide bonds	Rn00690933_m1	Probe spans exons	149
*Gapdh*	Glyceraldehyde‐3‐phosphate dehydrogenase	Enzyme participating in the glucose break down in the glycolysis pathway	Rn01462662_g1	Probe spans exons	90
*Hprt1*	Hypoxanthine phosphoribosyl transferase 1	Enzyme playing a central role in the generation of purine nucleotides	Rn01527840_m1	Probe spans exons	64
*Actb*	Actin, beta	Cytoskeletal protein nvolved in cellular motility, structure, integrity and signalling	Rn00667869_m1	Probe spans exons	91
*B2m*	Beta‐2 microglobulin	Component protein of MHC class I molecules	Rn00560865_m1	Probe spans exons	58

## 3 RESULTS

The presence of cerebral infarction was confirmed using TTC staining of coronal brain sections (Supplemental Material, Figure S1). Ninety minutes of tMCAO caused a large injury, which involved a major part of the DS and cerebral cortex. HIP was in the periinfarct zone.

**FIGURE 1:**
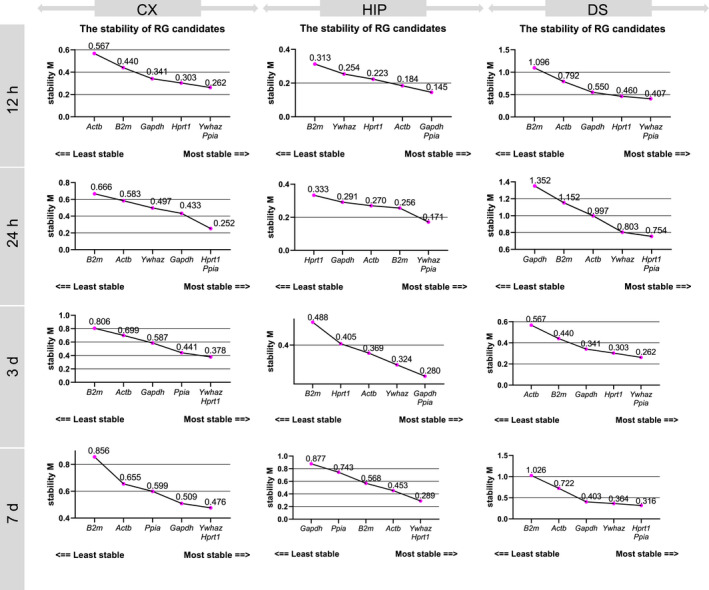
GeNorm analysis showing the stability of RG candidates in the cerebral cortex (CX), in the hippocampus (HIP) and in the dorsal striatum (DS) at four time points after tMCAO: 12 and 24 h; 3 and 7 days

Figure 1 and Table 2 present a comprehensive overview of the results of performed statistical analyses of the evaluated candidate RGs. The results of the variance analysis demonstrated that each gene showed significant variation in expression at a particular time point in the CX and DS, whereas in the HIP, only *Ppia* mRNA expression was stable across all time points (Supplemental Material, Figure S2).

**TABLE 2:** Stability ranking of candidate RGs in the ipsilateral cerebral cortex, hippocampus and dorsal striatum at four time points after tMCAO, 12 and 24 h; 3 and 7 days

	Rank	Comprehensive ranking		ΔCt		BestKeeper				NormFinder		GeNorm	
		Gene	Geomean	Gene	Average SD	Gene	SD (±Ct)	Cv (%Ct)	r	Gene	S	Gene	M
Cerebral cortex—12 h	1	*Ywhaz*	1.00	*Ywhaz*	0.45	*Ywhaz*	0.69	2.50	0.967	*Ywhaz*	0.151	*Ywhaz*	0.262
	2	*Ppia*	2.00	*Ppia*	0.46	*Ppia*	0.68	2.81	0.961	*Ppia*	0.181	*Ppia*	0.262
	3	*Hprt1*	3.22	*Gapdh*	0.50	*Hprt1*	0.80	2.86	0.958	*Hprt1*	0.295	*Hprt1*	0.303
	4	*Gapdh*	3.72	*Hprt1*	0.51	*Gapdh*	0.50	2.19	0.921	*Gapdh*	0.300	*Gapdh*	0.341
	5	*B2m*	5.00	*B2m*	0.66	***B2m***	**0.43**	**1.81**	**0.763**	*B2m*	0.550	*B2m*	0.440
	6	*Actb*	6.00	*Actb*	0.82	*Actb*	1.05	4.41	0.877	*Actb*	0.753	*Actb*	0.567
Cerebral cortex—24 h	1	*Ppia*	1.32	*Ppia*	0.55	*Ywhaz*	0.54	1.98	0.894	*Ppia*	0.224	*Ppia*	0.252
	2	*Hprt1*	2.38	*Hprt1*	0.57	*Gapdh*	0.58	2.50	0.830	*Hprt1*	0.284	*Hprt1*	0.252
	3	*Ywhaz*	2.45	*Ywhaz*	0.64	*Ppia*	0.33	1.47	0.728	*Ywhaz*	0.432	*Gapdh*	0.433
	4	*Gapdh*	3.50	*Actb*	0.69	*Hprt1*	0.32	1.23	0.650	*Actb*	0.501	*Ywhaz*	0.497
	5	*Actb*	4.47	*Gapdh*	0.71	*Actb*	0.40	1.69	0.456	*Gapdh*	0.586	*Actb*	0.583
	6	*B2m*	6.00	*B2m*	0.83	*B2m*	0.42	1.88	0.001	*B2m*	0.738	*B2m*	0.666
Cerebral cortex—3 days	1	*Ppia*	1.32	*Ppia*	0.65	*Ppia*	0.98	4.46	0.974	*Ppia*	0.109	*Ywhaz*	0.372
	2	*Ywhaz*	1.68	*Ywhaz*	0.69	*Ywhaz*	0.92	3.62	0.934	*Ywhaz*	0.367	*Hprt1*	0.372
	3	*Hprt1*	2.71	*Hprt1*	0.70	*Hprt1*	0.81	3.16	0.925	*Hprt1*	0.391	*Ppia*	0.434
	4	*Gapdh*	4.47	*Actb*	0.80	*Gapdh*	0.63	2.90	0.745	*Actb*	0.541	*Gapdh*	0.584
	5	*Actb*	4.68	*Gapdh*	0.95	***Actb***	**1.13**	**5.20**	**0.939**	***Gapdh***	0.841	*Actb*	0.691
	6	*B2m*	6.00	*B2m*	1.03	***B2m***	**1.46**	**6.70**	**0.931**	***B2m***	0.945	*B2m*	0.804
Cerebral cortex—7 days	1	*Hprt1*	1.00	*Hprt1*	0.65	*Hprt1*	0.37	1.54	0.873	*Hprt1*	0.221	*Hprt1*	0.477
	2	*Ppia*	2.63	*Ppia*	0.76	*Actb*	0.66	3.28	0.851	*Ppia*	0.365	*Ywhaz*	0.477
	3	*Actb*	3.08	*Actb*	0.81	*Ppia*	0.42	2.06	0.714	*Actb*	0.463	*Gapdh*	0.509
	4	*Ywhaz*	3.36	*Ywhaz*	0.81	*Ywhaz*	0.49	2.09	0.496	*Ywhaz*	0.603	*Ppia*	0.599
	5	*Gapdh*	4.40	*Gapdh*	0.85	*Gapdh*	0.47	2.25	0.338	*Gapdh*	0.665	*Actb*	0.656
	6	*B2m*	6.00	*B2m*	1.25	***B2m***	**1.22**	**6.04**	**0.838**	***B2m***	1.179	*B2m*	0.855
Hippocampus—12 h	1	*Ppia*	1.19	*Ppia*	0.25	*Actb*	0.28	1.42	0.933	*Ppia*	0.076	*Ppia*	0.144
	2	*Gapdh*	2.21	*Gapdh*	0.26	*Ppia*	0.18	0.90	0.872	*Gapdh*	0.107	*Gapdh*	0.144
	3	*Actb*	2.28	*Actb*	0.27	*Gapdh*	0.20	1.02	0.856	*Actb*	0.156	*Actb*	0.182
	4	*Hprt1*	4.23	*Hprt1*	0.31	*Ywhaz*	0.26	1.26	0.671	*Hprt1*	0.213	*Hprt1*	0.221
	5	*Ywhaz*	4.73	*Ywhaz*	0.35	*Hprt1*	0.17	0.78	0.495	*Ywhaz*	0.292	*Ywhaz*	0.254
	6	*B2m*	6.00	*B2m*	0.43	*B2m*	0.26	1.25	0.138	*B2m*	0.394	*B2m*	0.313
Hippocampus—24 h	1	*Ppia*	1.68	*B2m*	0.29	*Ywhaz*	0.35	1.71	0.896	*B2m*	0.144	*Ppia*	0.168
	2	*B2m*	1.73	*Ppia*	0.30	*Ppia*	0.33	1.62	0.884	*Ppia*	0.177	*Ywhaz*	0.168
	3	*Ywhaz*	2.06	*Ywhaz*	0.31	*B2m*	0.26	1.25	0.857	*Ywhaz*	0.194	*B2m*	0.254
	4	*Actb*	4.23	*Actb*	0.32	*Gapdh*	0.39	1.99	0.848	*Actb*	0.215	*Actb*	0.268
	5	*Gapdh*	4.73	*Gapdh*	0.36	*Actb*	0.34	1.67	0.844	*Gapdh*	0.301	*Gapdh*	0.289
	6	*Hprt1*	6.00	*Hprt1*	0.42	*Hprt1*	0.22	0.96	0.385	*Hprt1*	0.368	*Hprt1*	0.332
Hippocampus—3 days	1	*Gapdh*	1.00	*Gapdh*	0.37	*Gapdh*	0.28	1.44	0.960	*Gapdh*	0.139	*Gapdh*	0.279
	2	*Ywhaz*	2.91	*Ywhaz*	0.45	*Hprt1*	0.65	2.80	0.896	*Ppia*	0.274	*Ppia*	0.279
	3	*Ppia*	2.99	*Actb*	0.45	*Ywhaz*	0.45	2.15	0.893	*Actb*	0.277	*Ywhaz*	0.325
	4	*Actb*	3.46	*Ppia*	0.46	*Actb*	0.47	2.24	0.890	*Ywhaz*	0.278	*Actb*	0.370
	5	*Hprt1*	3.97	*Hprt1*	0.54	*Ppia*	0.36	1.73	0.778	*Hprt1*	0.464	*Hprt1*	0.405
	6	*B2m*	6.00	*B2m*	0.66	*B2m*	0.32	1.48	0.131	*B2m*	0.603	*B2m*	0.489
Hippocampus—7 days	1	*Ywhaz*	1.00	*Ywhaz*	0.66	*Ywhaz*	0.51	2.26	0.946	*Ywhaz*	0.146	*Ywhaz*	0.293
	2	*Hprt1*	2.00	*Hprt1*	0.69	*Hprt1*	0.63	2.55	0.914	*Hprt1*	0.207	*Hprt1*	0.293
	3	*Actb*	3.00	*Actb*	0.84	*Actb*	0.82	3.58	0.826	*Actb*	0.557	*Actb*	0.459
	4	*B2m*	4.00	*B2m*	0.85	*B2m*	0.78	3.44	0.825	*B2m*	0.572	*B2m*	0.572
	5	*Ppia*	5.23	*Ppia*	1.10	*Gapdh*	0.68	3.17	0.501	*Ppia*	0.954	*Ppia*	0.746
	6	*Gapdh*	5.73	*Gapdh*	1.16	*Ppia*	0.68	3.00	0.364	*Gapdh*	1.038	*Gapdh*	0.883
Dorsal Striatum—12 h	1	*Hprt1*	1.32	*Hprt1*	0.81	*Hprt1*	0.28	1.10	0.809	*Hprt1*	0.206	*Ppia*	0.406
	2	*Ppia*	1.68	*Ppia*	0.90	*Ppia*	0.58	2.56	0.722	*Ppia*	0.377	*Ywhaz*	0.406
	3	*Ywhaz*	2.91	*Ywhaz*	0.90	*Actb*	0.80	3.72	0.511	*Ywhaz*	0.450	*Hprt1*	0.460
	4	*Gapdh*	4.23	*Gapdh*	1.00	*Ywhaz*	0.52	2.28	0.553	*Gapdh*	0.623	*Gapdh*	0.550
	5	*Actb*	4.40	*Actb*	1.27	*Gapdh*	0.33	1.59	0.129	*Actb*	1.064	*Actb*	0.792
	6	*B2m*	4.73	*B2m*	1.71	***B2m***	**1.43**	**6.25**	**0.823**	*B2m*	1.602	*B2m*	1.097
Dorsal Striatum—24 h	1	*Ppia*	1.00	*Ppia*	0.91	*Ppia*	0.70	3.17	0.938	*Ppia*	0.201	*Ppia*	0.402
	2	*Hprt1*	2.21	*Hprt1*	1.00	*Ywhaz*	0.91	4.05	0.851	*Hprt1*	0.464	*Hprt1*	0.402
	3	*Ywhaz*	2.71	*Ywhaz*	1.04	*Hprt1*	0.56	2.23	0.832	*Ywhaz*	0.525	*Ywhaz*	0.632
	4	*B2m*	4.42	*B2m*	1.25	*Actb*	0.61	2.99	0.209	*B2m*	0.929	*B2m*	0.865
	5	*Gapdh*	5.00	*Gapdh*	1.46	***Gapdh***	**1.86**	**8.53**	**0.969**	*Gapdh*	1.273	*Gapdh*	1.040
	6	*Actb*	5.42	*Actb*	1.52	***B2m***	**1.20**	**5.34**	**0.867**	*Actb*	1.369	*Actb*	1.199
Dorsal Striatum—3 days	1	*Gapdh*	1.32	*Gapdh*	0.72	*Gapdh*	0.68	3.41	0.897	*Gapdh*	0.389	*Ywhaz*	0.286
	2	*Hprt1*	2.00	*Hprt1*	0.73	*Hprt1*	0.92	3.76	0.897	*Hprt1*	0.483	*Hprt1*	0.286
	3	*Ywhaz*	2.28	*Ywhaz*	0.74	*Ywhaz*	0.74	3.40	0.841	*Ywhaz*	0.522	*Gapdh*	0.415
	4	*B2m*	4.23	*B2m*	0.78	*Ppia*	0.49	2.41	0.836	*B2m*	0.529	*B2m*	0.626
	5	*Actb*	5.23	*Actb*	0.89	***B2m***	**1.06**	**5.27**	**0.937**	*Actb*	0.723	*Actb*	0.716
	6	*Ppia*	5.42	*Ppia*	0.99	***Actb***	**1.18**	**6.22**	**0.923**	*Ppia*	0.834	*Ppia*	0.809
Dorsal Striatum—7 days	1	*Ppia*	1.00	*Ppia*	0.77	*Ppia*	0.25	1.22	0.867	*Ppia*	0.170	*Ppia*	0.314
	2	*Hprt1*	2.00	*Hprt1*	0.79	*Hprt1*	0.35	1.44	0.736	*Hprt1*	0.278	*Hprt1*	0.314
	3	*Ywhaz*	3.00	*Ywhaz*	0.89	*Ywhaz*	0.34	1.56	0.184	*Ywhaz*	0.709	*Ywhaz*	0.364
	4	*Gapdh*	4.00	*Gapdh*	0.93	*Gapdh*	0.27	1.33	0.043	*Gapdh*	0.739	*Gapdh*	0.404
	5	*Actb*	5.23	*Actb*	1.12	***B2m***	**1.74**	**9.04**	**0.955**	*Actb*	0.761	*Actb*	0.725
	6	*B2m*	5.73	*B2m*	1.61	***Actb***	**1.17**	**6.27**	**0.952**	*B2m*	1.564	*B2m*	1.020

Note

The comprehensive ranking was calculated by geometric mean method of rank positions for each gene in each statistical method ‐ ΔCt, BestKeeper, NormFinder and GeNorm. According to this approach, *Ppia* was the most stable gene in the rat model of tMCAO (*Ppia* > *Hprt1* > *Ywhaz* > *Gapdh* > *Actb* > *B2m*). In the ΔCt method the determinant of gene stability was the mean ± SD of the expression of the particular RG. Using this method, the following order of gene stability across all brain structures and time points after tMCAO was obtained: *Ppia* > *Hprt1* > *Ywhaz* > *Gapdh* > *Actb* > *B2m*. BestKeeper analysis uses standard deviations (SDs), coefficients of variance (CVs), and Pearson correlation coefficients (rs) to rank analysed genes according to stability. In this analysis, the order of the genes according to stability was as following: *Ywhaz* > *Ppia* > *Hprt1* > *Gapdh* > *Actb* > *B2m*. Because Actb had a high SD was classified as unstable and excluded in the analysis for the CX 12 h, DS 3 days and 7 days. For the same reasons, B2m was excluded from the analysis for the CX at 3 and 7 days and for the DS at all timepoints, and *Gapdh* was excluded from the analysis for the DS at 24 h after tMCAO. The NormFinder expresses the stability measure as the S. The lowest S denotes the most stable gene. The NormFinder analysis identified *Ppia* as the most stable gene across analysed brain structures and time points after reperfusion. However, in the DS at 3 days after tMCAO, *Ppia*, which had an S value of 0.834, was the least stable RG, and *Gapdh*, which had an S value of 0.389, was the most stable RG. The GeNorm algorithm is based on pairwise variation, provides a stability value M. Genes with M values higher than 1.0 are considered unstable. The M values reached this cut‐off in DS 12 and 24 h; 7 days for genes such as *B2m*, *Actb*, *Gapdh*. The algorithm is based on the identification of two genes that are the most conserved in expression in a specific experimental setup. In the next step, the values of the rest of the genes are calculated in relation to the pair of most stable genes. Thus, the ranked list always starts with the two most stable genes with identical M values and proceeds with more unstable genes with increasing M values. The GeNorm comprehensive ranked list of stability, which was based on the geometric mean of ranks of this analysis for a particular brain area and time point, was as follows: *Ppia* >Ywhaz > *Hprt1* > *Gapdh* >B2m > *Actb*.

Bold values indicates statistical siginificance at *p* < 0.05

The GeNorm comprehensive ranked list of stability, which was based on the geometric mean of ranks of this analysis for a particular brain area and time point, was as follows: *Ppia*>Ywhaz>*Hprt1*>*Gapdh*>B2m>*Actb* (Table 2; Figure 1). The GeNorm pairwise variation value (V_n/n+1_) revealed that two to three RGs was an optimal number in this animal model to perform the reliable normalization. However, in the DS, which is the region that is most severely affected by ischaemia, more RGs were needed to normalize target gene expression (Supplemental Material, Figure S3).

Next, NormFinder analysis was performed and the gene ranking according to geometric mean was as follows: *Ppia*>Hprt1>*Ywhaz*>Gapdh>*Actb*>B2m (Table 2).

BestKeeper analysis showed that stability was as following: *Ywhaz*>*Ppia*>*Hprt1*>*Gapdh*>*Actb*>*B2m*.

In ΔCt method the gene stability after tMCAO was as following: *Ppia*>Hprt1>*Ywhaz*>Gapdh>*Actb*>B2m (Table 2).

The use of a geometric mean allowed us to present an integrated ranked list of gene stability across all the studied brain structures and time points. According to this approach, *Ppia* was the most stable gene in the rat model of tMCAO (*Ppia*>Hprt1>*Ywhaz*>Gapdh>*Actb*>B2m) (Table 2).

## 4 DISCUSSION

According to the majority of statistical methods applied, *Ppia* appeared to be the most promising RG. Two other genes, namely *Ywhaz* and *Hprt1*, might serve as complements for multigene normalization of RT‐qPCR results. *Gapdh* analysis results in some conflicting results, whereas *Actb* and *B2m* appeared to be highly unstable at the studied time points and regions.

Ischaemia and reperfusion initiate a series of pathological events with clear spatiotemporal diversity. Glutamate excitotoxicity, oxidative stress, mitochondrial dysfunction and neuroinflammation initiate several different mechanisms of cell death. The intensity of these events varies according to time point and brain region. Additionally, the cellular composition and molecular characteristics of affected regions change over time, with dying neurons, proliferating glia, invading peripheral inflammatory cells through the disrupted blood‐brain barrier. All these processes may influence the stability of potential RGs.

*Actb* was previously suggested as an unstable gene for RT‐qPCR data normalization in brain trauma and in both transient and permanent models of MCAO due to its variability in expression.^7^, ^8^, ^9^ Here, in the CX and DS at delayed time points (3 and 7 days), *Actb* expression was increased 4–6‐fold (Supplemental Material, Figure S2). This may be related to the proliferation of microglia, neutrophils/monocytes and activated astrocytes developing extensive processes within the infarcted brain area.

*B2m* encodes a small protein (11.8 kDa) that is a component of the major histocompatibility class I (MHC I) complex. The role of B2m protein in cerebral ischaemia is unclear; however, its serum levels are associated with an increased risk of ischaemic stroke in humans.^10^ Neuroinflammation plays a crucial role after brain ischaemia. A lack of MHC I in animals subjected to focal cerebral ischaemia is neuroprotective.^11^ The instability of *B2m* was reported in a global brain ischaemia model.^12^ This suggests that in cerebral ischaemia, the mRNA expression of *B2m* might be regulated. In the present study, at 3 and 7 days after tMCAO, the expression of *B2m* was increased 5–10‐fold in the most affected brain structures.

*Gapdh* was reported to be an unstable gene in stroke models.^13^, ^14^, ^15^ The expression of protein product in a model of tMCAO correlates with its proapoptotic action.^16^ Here, in the HIP and DS at 3 days after reperfusion, *Gapdh* was ranked as the most stable gene, but in the DS at 24 h, this gene was excluded from analysis due to its high SD value. Apoptosis occurs with some delay after ischaemic injury, which corresponds to increased expression of *Gapdh* at later time points (Supplemental Material, Figure S2).

In the current study, next to *Ppia*, *Hprt1* and *Ywhaz* were the most reliable and stable genes in the infarct area (the DS and CX) at every studied time point (Table 2; Figure 1). This is in line with previous reports on an MCAO model; however, it is worth to mention that *Ppia* in one report was unstable but in a model of permanent MCAO.^7^, ^15^ *Hprt1* is a stable RG in mice with permanent and transient MCAO.^7^, ^17^ *Ywhaz* and *Hprt1* have been reported to be suitable RGs in rats with pMCAO.^15^ However, to date, there have been no reports on their stability in a rat model of tMCAO. HPRT is an enzyme catalysing the synthesis of energy‐rich purines. In stroke models its activity is unaffected.^18^ The role of protein 14‐3‐3, which is encoded by *Ywhaz*, in cerebral ischaemia is unknown. This protein is predominantly expressed in the brain. Some data suggest that the 14‐3‐3 protein regulates NFκB. This signalling pathway is essential for cell survival, including in stroke, and the 14‐3‐3 protein might be a therapeutic target for neurological diseases.^19^ *Ppia* encodes cyclophilin A, a protein that interacts with apoptosis inducible factor and mediates the activation of caspase‐independent apoptosis in ischaemia‐reperfusion injury.^20^ In this study, tMCAO model of cerebral ischaemia was used. In this model, there are two critical moments—occlusion of the middle cerebral artery, and after 90 min its reperfusion. The restoration of perfusion initiates other mechanisms of cellular degeneration. This may explain that in permanent ischaemia *Ppia* is unstable, but in contrast to ischaemia which is followed by reperfusion.^15^

To conclude, this study provided a clear guide on the selection of RGs appropriate for particular brain structures and time points after tMCAO. This study indicate that *Ppia* is the most reliable RG candidate in a rat tMCAO model, whereas *Hprt1* and *Ywhaz* were found to be complementary RGs. As shown, at the delayed time point after reperfusion, such as 3 or 7 days there are no perfect RGs, here we suggest that RT‐qPCR results should be normalized not with just one RGs, but specifically for CX and DS, with at least three most stable RGs. These should definitely standardize the analysis at different time points and in different brain areas more or less associated with ischaemic core or penumbra area. This study confirms that for every experimental setup, verifying RGs stability is strongly recommended and presented here analysis can be used as a guidance. To the best of our knowledge, there are no detailed data on the expression of RGs at various time points after reperfusion in various brains areas in a rat model of tMCAO. These data fill the existing knowledge gap and thus improve research on target gene expression using RT‐qPCR.

## CONFLICT OF INTEREST

The authors declare that they have no conflict of interest.

## AUTHOR CONTRIBUTIONS

**Bartosz Pomierny:** Conceptualization (lead); Data curation (equal); Formal analysis (equal); Funding acquisition (lead); Investigation (equal); Methodology (equal); Project administration (lead); Visualization (lead); Writing – original draft (lead). **Weronika Krzyżanowska:** Conceptualization (equal); Data curation (equal); Formal analysis (equal); Investigation (equal); Methodology (equal); Validation (equal); Writing – original draft (equal). **Jakub Jurczyk:** Data curation (equal); Investigation (equal); Methodology (equal); Validation (equal); Writing – original draft (equal). **Beata Strach:** Data curation (equal); Investigation (equal); Methodology (equal); Writing – original draft (equal). **Alicja Skórkowska:** Data curation (equal); Investigation (equal); Methodology (equal); Writing – original draft (equal). **Innesa Leonovich:** Data curation (equal); Investigation (equal); Methodology (equal). **Bogusława Budziszewska:** Conceptualization (equal); Investigation (equal); Writing – original draft (equal). **Joanna Pera:** Conceptualization (lead); Funding acquisition (lead); Investigation (equal); Methodology (equal); Project administration (lead); Supervision (lead); Writing – original draft (lead).

## CONSENT FOR PUBLICATION

All authors have given final approval of the version and agreed with the publication of this study here.

## Supporting information

Figure S1

Figure S2

Figure S3

Supplementary Material

## References

[1] Andersen CL , Jensen JL , Ørntoft TF . Normalization of real‐time quantitative reverse transcription‐PCR data: a model‐based variance estimation approach to identify genes suited for normalization, applied to bladder and colon cancer data sets. Cancer Res. 2004;64(15):5245‐5250. doi:10.1158/0008-5472.CAN-04-0496 15289330

[2] Vandesompele J , De Preter K , Pattyn F , et al. Accurate normalization of real‐time quantitative RT‐PCR data by geometric averaging of multiple internal control genes. Genome Biol. 2002;3:RESEARCH0034. 12184808 10.1186/gb-2002-3-7-research0034 PMC126239

[3] Livak KJ , Schmittgen TD . Analysis of relative gene expression data using real‐time quantitative PCR and the 2‐ΔΔCT method. Methods. 2001;25:402‐408. 11846609 10.1006/meth.2001.1262

[4] Pfaffl MW , Tichopad A , Prgomet C , Neuvians TP . Determination of stable housekeeping genes, differentially regulated target genes and sample integrity: BestKeeper ‐ Excel‐based tool using pair‐wise correlations. Biotechnol Lett. 2004;26:509‐515. 15127793 10.1023/b:bile.0000019559.84305.47

[5] Silver N , Best S , Jiang J , Thein SL . Selection of housekeeping genes for gene expression studies in human reticulocytes using real‐time PCR. BMC Mol Biol. 2006;7:33. 17026756 10.1186/1471-2199-7-33 PMC1609175

[6] Krzyżanowska W , Pomierny B , Budziszewska B , et al. Ceftriaxone‐ and N‐acetylcysteine‐induced brain tolerance to ischemia: influence on glutamate levels in focal cerebral ischemia. PLoS One. 2017;12(10):e0186243. 29045497 10.1371/journal.pone.0186243 PMC5646803

[7] Kang Y , Wu Z , Cai D , Lu B . Evaluation of reference genes for gene expression studies in mouse and N2a cell ischemic stroke models using quantitative real‐time PCR. BMC Neurosci. 2018;19:3. 29390963 10.1186/s12868-018-0403-6 PMC5795833

[8] Harrison DC , Medhurst AD , Bond BC , Campbell CA , Davis RP , Philpott KL . The use of quantitative RT‐PCR to measure mRNA expression in a rat model of focal ischemia ‐ Caspase‐3 as a case study. Mol Brain Res. 2000;75:143‐149. 10648898 10.1016/s0169-328x(99)00305-8

[9] Tian YF , Zhang PB , Xiao XL , et al. The quantification of ADAMTS expression in an animal model of cerebral ischemia using real‐time PCR. Acta Anaesthesiol Scand. 2007;51:158‐164. 17073862 10.1111/j.1399-6576.2006.01161.x

[10] Qun S , Hu F , Wang G , et al. Serum beta2‐microglobulin levels are highly associated with the risk of acute ischemic stroke. Sci Rep. 2019;9:6883. 31053801 10.1038/s41598-019-43370-9 PMC6499788

[11] Adelson JD , Barreto GE , Xu L , et al. Neuroprotection from stroke in the absence of MHCI or PirB. Neuron. 2012;73:1100‐1107. 22445338 10.1016/j.neuron.2012.01.020 PMC3314229

[12] Langnaese K , John R , Schweizer H , Ebmeyer U , Keilhoff G . Selection of reference genes for quantitative real‐time PCR in a rat asphyxial cardiac arrest model. BMC Mol Biol. 2008;9(1):53. 18505597 10.1186/1471-2199-9-53 PMC2430208

[13] Nishida Y , Sugahara‐Kobayashi M , Takahashi Y , Nagata T , Ishikawa K , Asai S . Screening for control genes in mouse hippocampus after transient forebrain ischemia using high‐density oligonucleotide array. J Pharmacol Sci. 2006;101:52‐57. 16717400 10.1254/jphs.fp0050881

[14] Kobayashi MS , Takahashi Y , Nagata T , et al. Screening for control genes in rat global cerebral ischemia using high‐density oligonucleotide array. J Neurosci Res. 2004;76:512‐518. 15114623 10.1002/jnr.20094

[15] Gubern C , Hurtado O , Rodríguez R , et al. Validation of housekeeping genes for quantitative real‐time PCR in in‐vivo and in‐vitro models of cerebral ischaemia. BMC Mol Biol. 2009;10:57. 19531214 10.1186/1471-2199-10-57 PMC2706836

[16] Tanaka R , Mochizuki H , Suzuki A , et al. Induction of Glyceraldehyde‐3‐Phosphate Dehydrogenase (GAPDH) expression in rat brain after focal ischemia/reperfusion. J Cereb Blood Flow Metab. 2002;22:280‐288. 11891433 10.1097/00004647-200203000-00005

[17] Meldgaard M , Fenger C , Lambertsen KL , Pedersen MD , Ladeby R , Finsen B . Validation of two reference genes for mRNA level studies of murine disease models in neurobiology. J Neurosci Methods. 2006;156:101‐110. 16554095 10.1016/j.jneumeth.2006.02.008

[18] Cherin T , Catbagan M , Treiman S , Mink R . The effect of normothermic and hypothermic hypoxia‐ischemia on brain hypoxanthine phosphoribosyl transferase activity. Neurol Res. 2006;28:831‐836. 17288740 10.1179/016164105X49229

[19] Zhou X‐Y , Hu DX , Chen RQ , Chen XQ , Dong W‐L , Yi C‐L . 14‐3‐3 isoforms differentially regulate NFκB signaling in the brain after ischemia‐reperfusion. Neurochem Res. 2017;42:2354‐2362. 28424948 10.1007/s11064-017-2255-3

[20] Rodriguez J , Xie C , Li T , et al. Inhibiting the interaction between apoptosis‐inducing factor and cyclophilin A prevents brain injury in neonatal mice after hypoxia‐ischemia. Neuropharmacology. 2020;171:108088. 32277944 10.1016/j.neuropharm.2020.108088

