# Supplementary figures and images for: Identification of optimal reference genes for gene expression studies in a focal cerebral ischaemia model—Spatiotemporal effects
Source: J Cell Mol Med. 2022 Apr 22;26(10):3060–7. doi: 10.1111/jcmm.17284 (PMC9097850; doi:10.1111/jcmm.17284)

SHAM

tMCAO

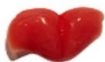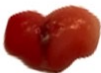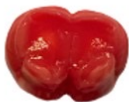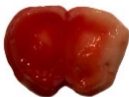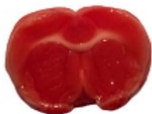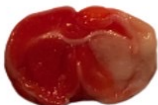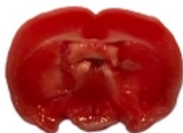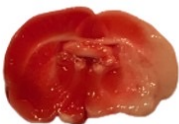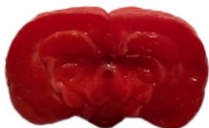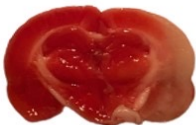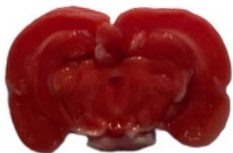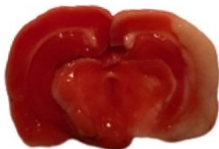

Supplement: Supplementary file 1 — Figure S1 [file JCMM-26-3060-s004.pdf]

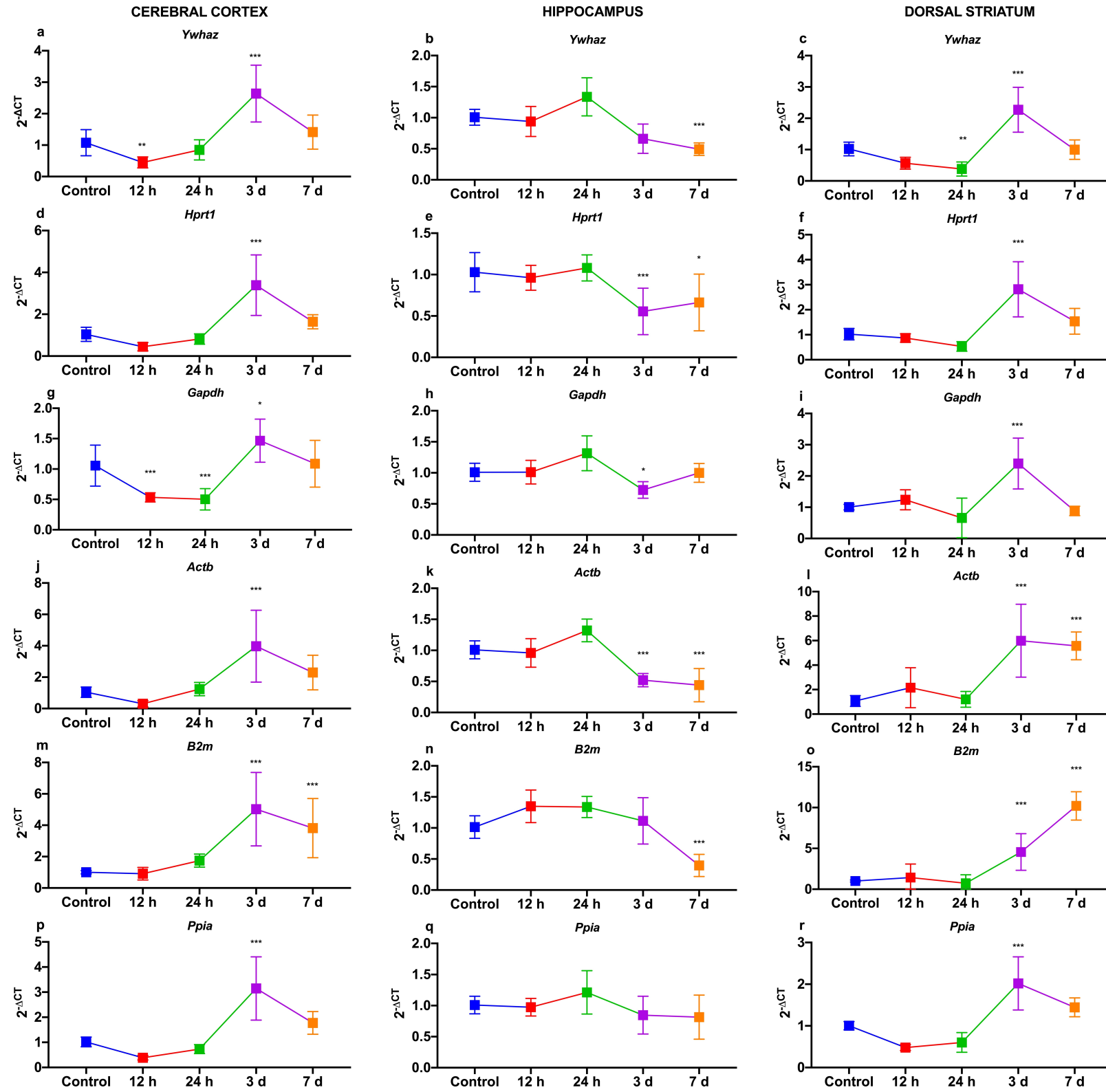

Supplement: Supplementary file 2 — Figure S2 [file JCMM-26-3060-s002.pdf]

CX

HIP

DS

12 h

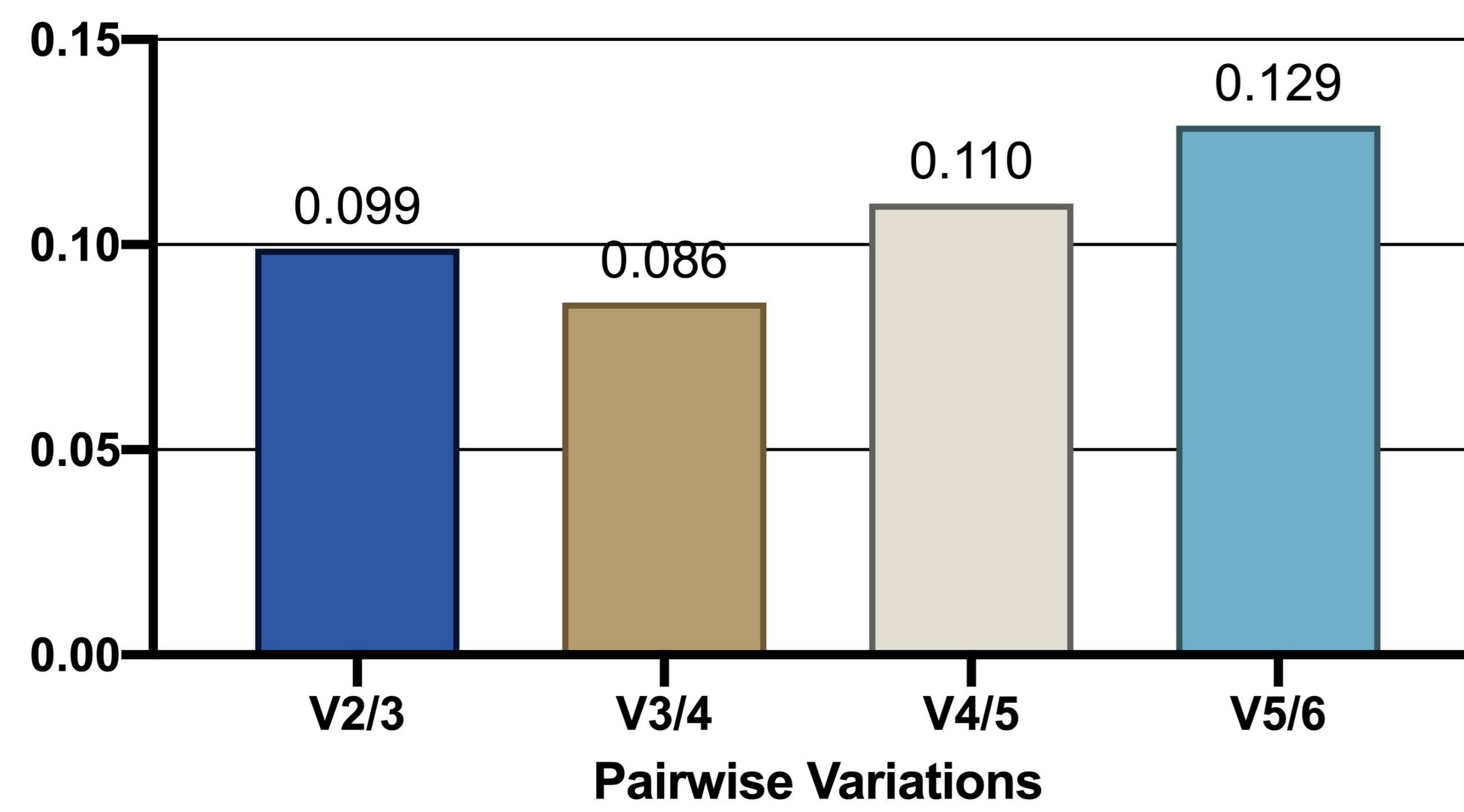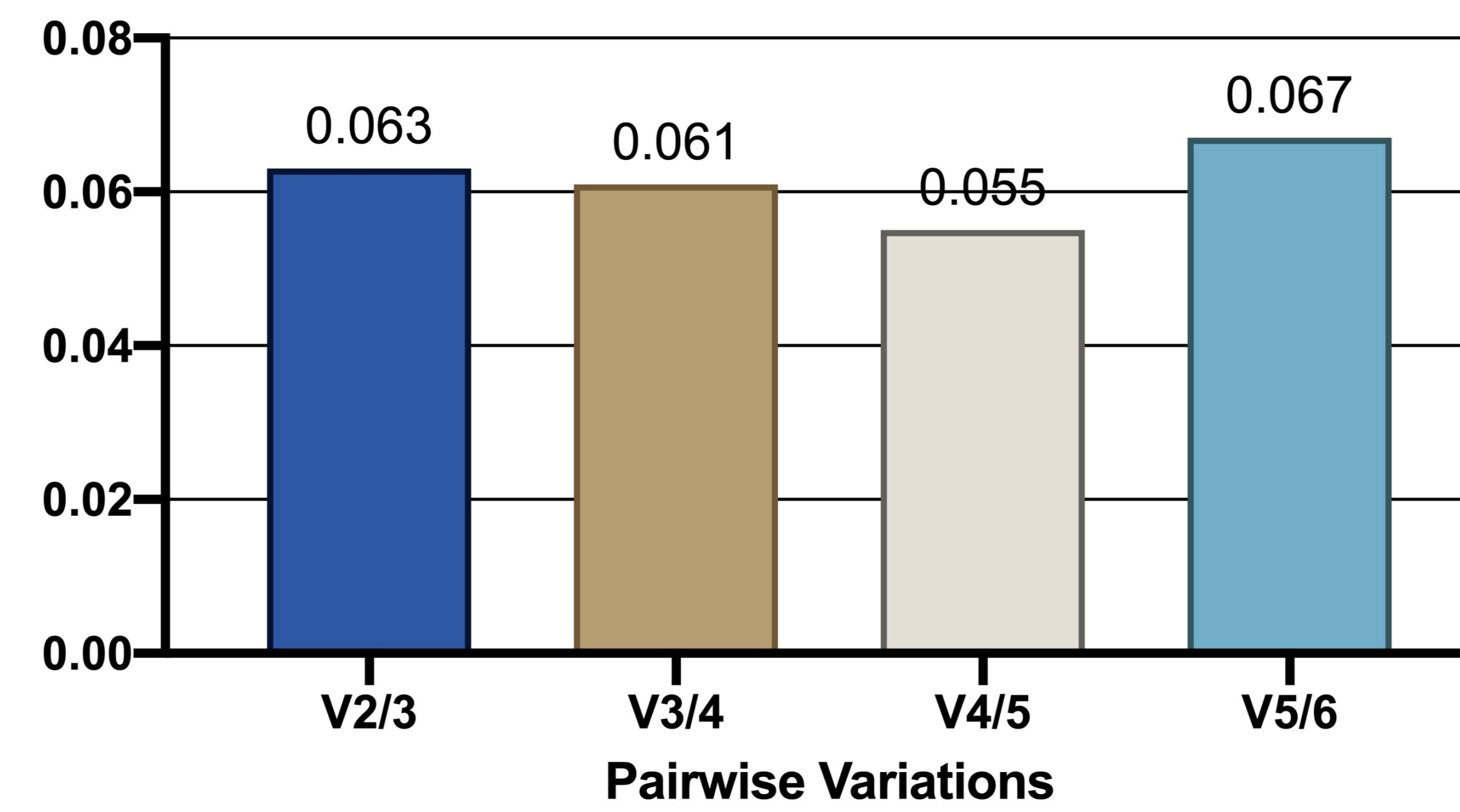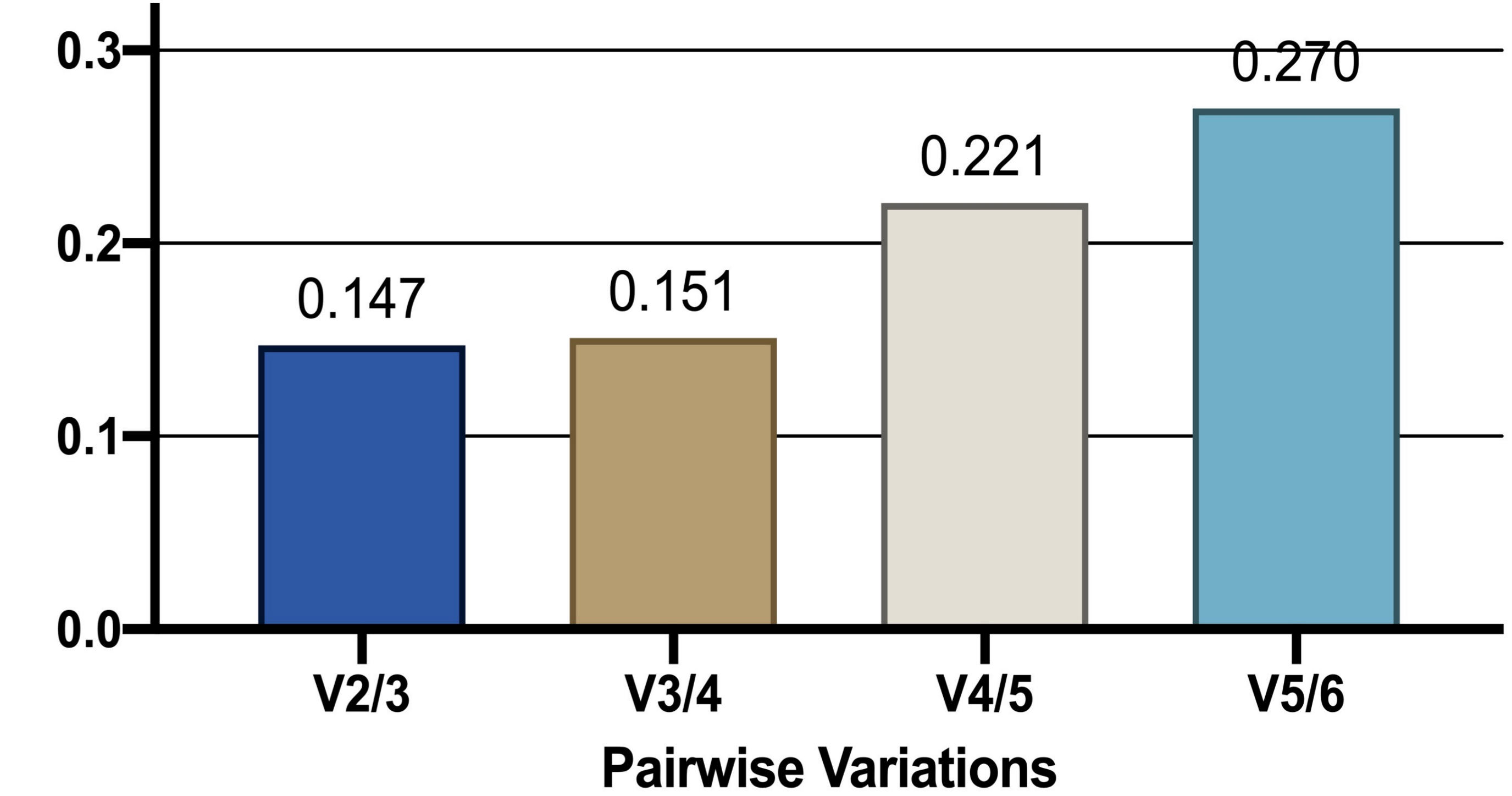

24 h

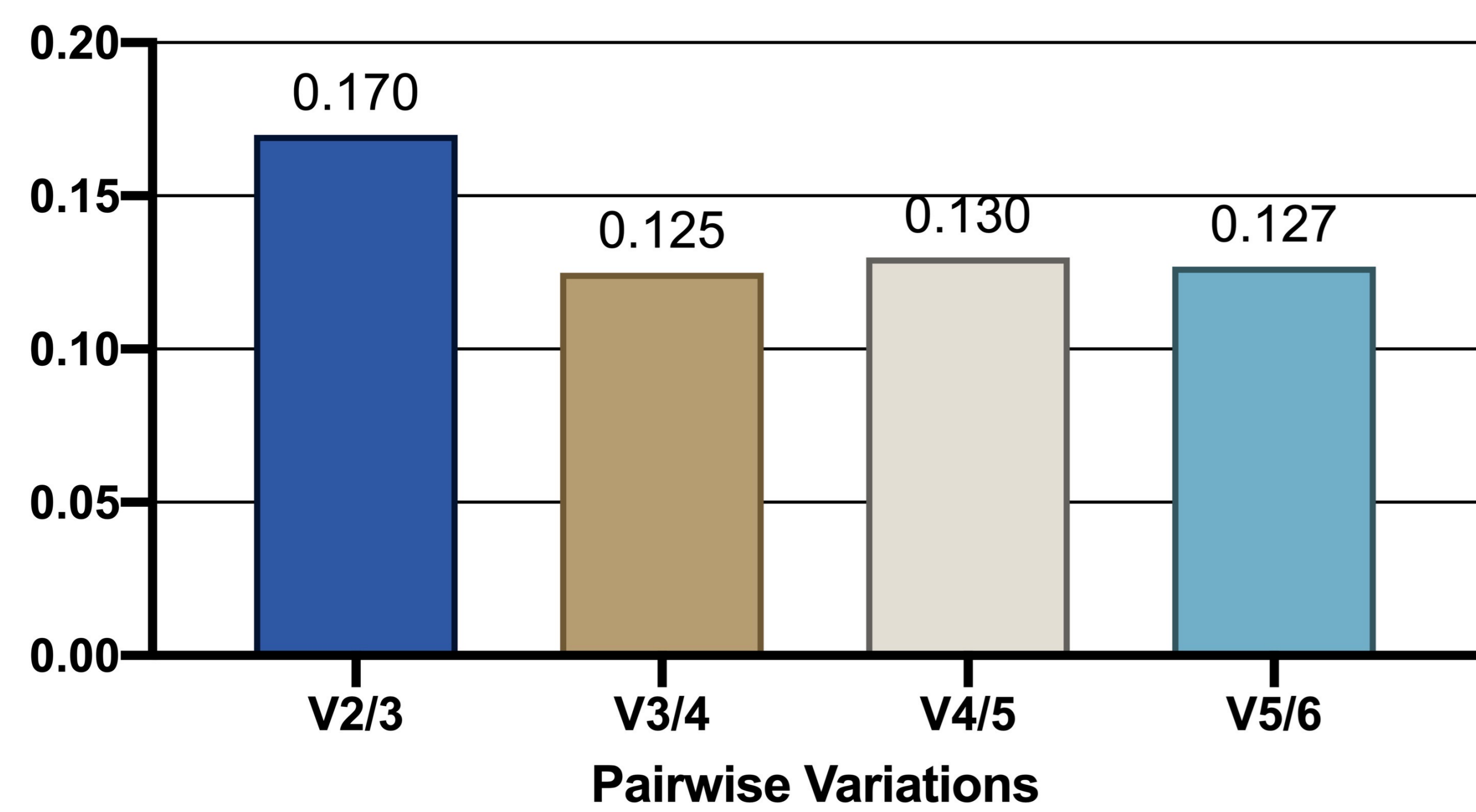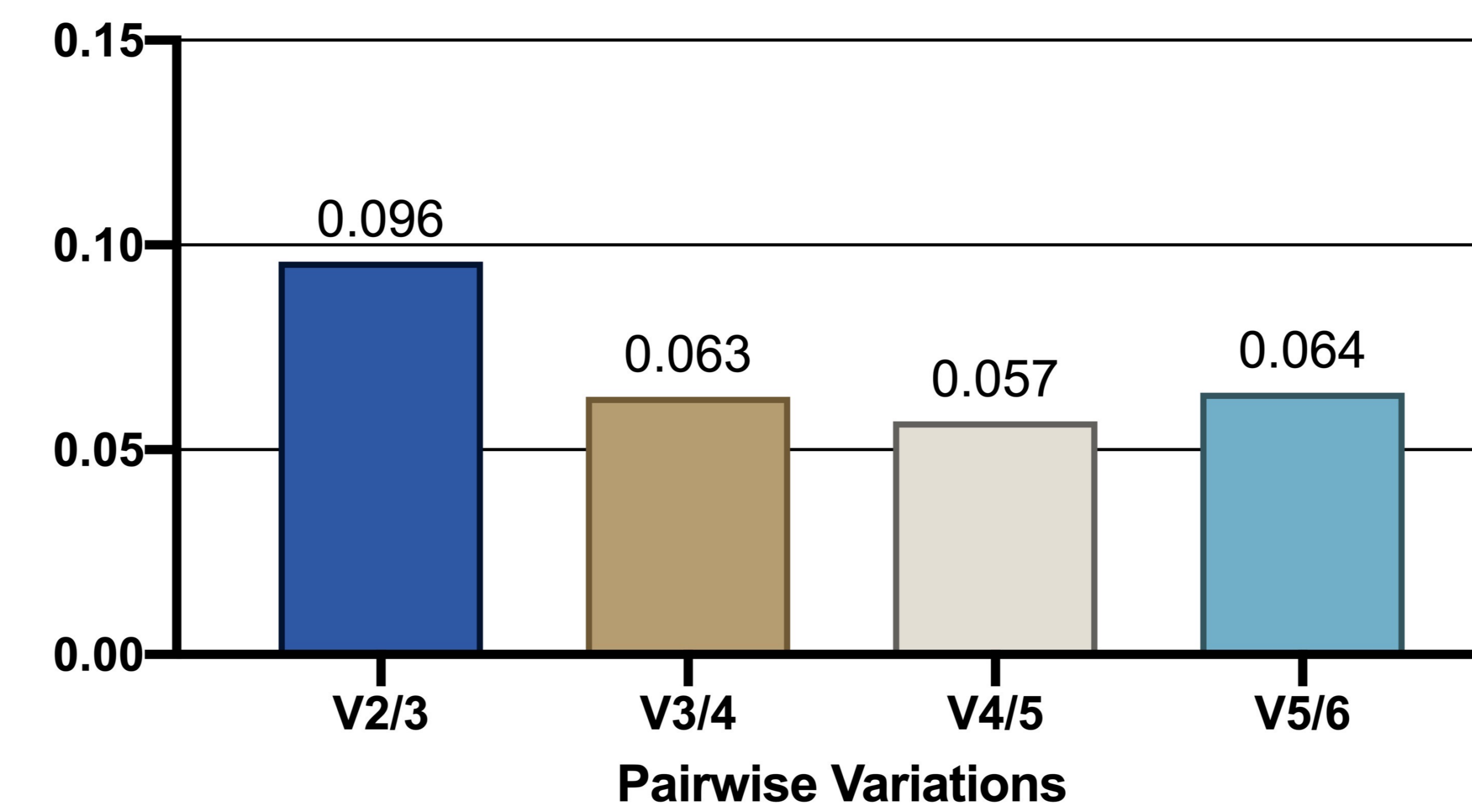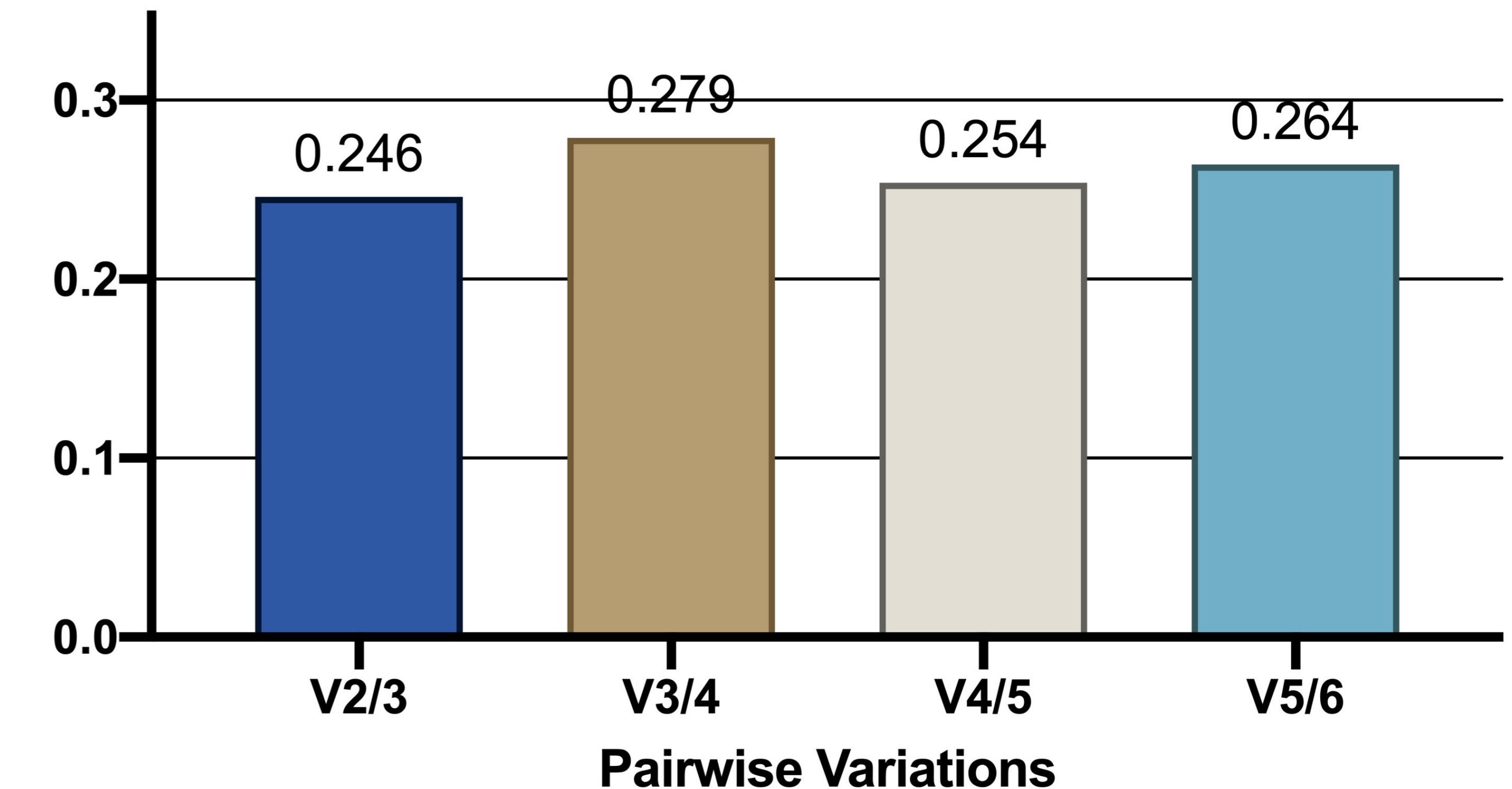

3 d

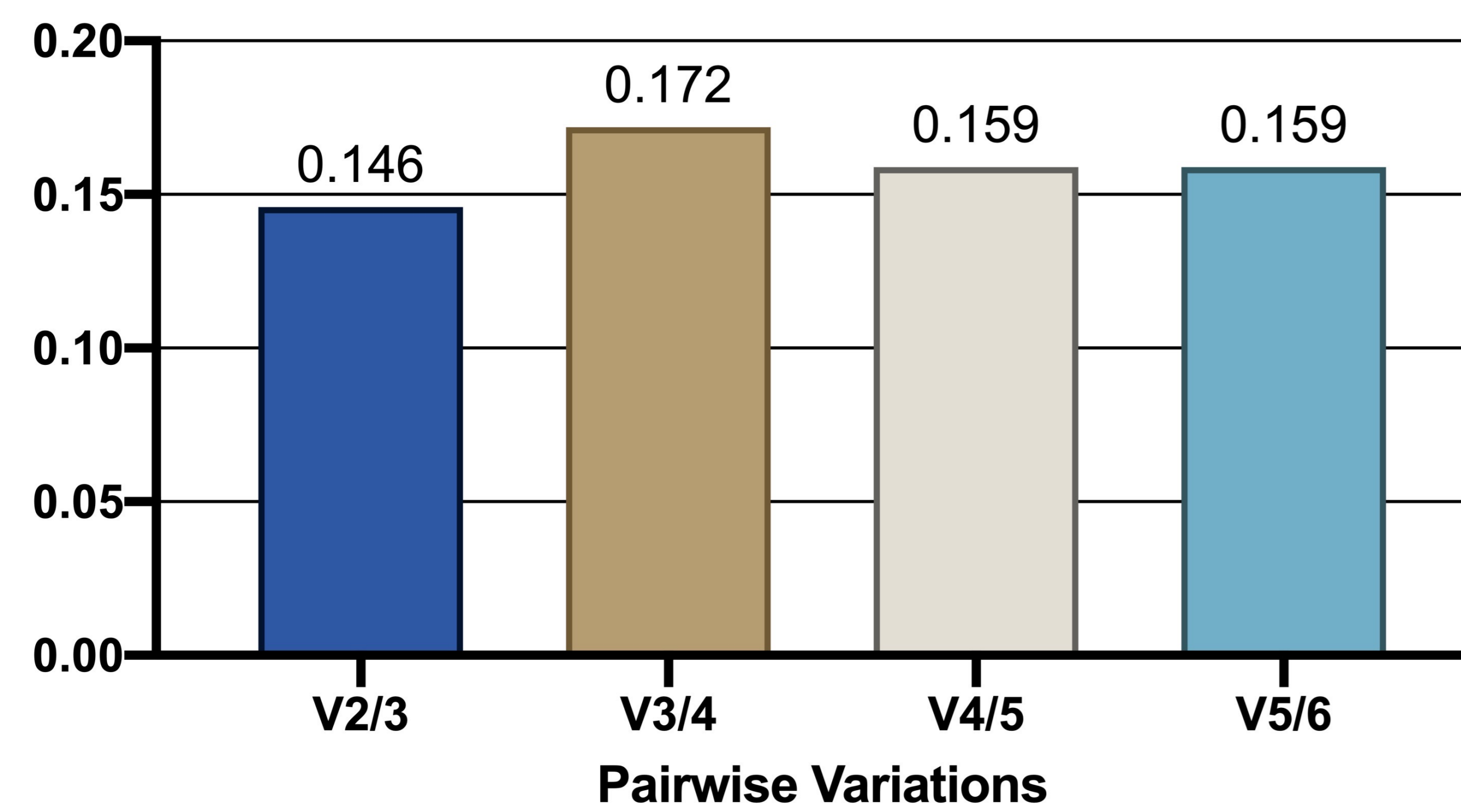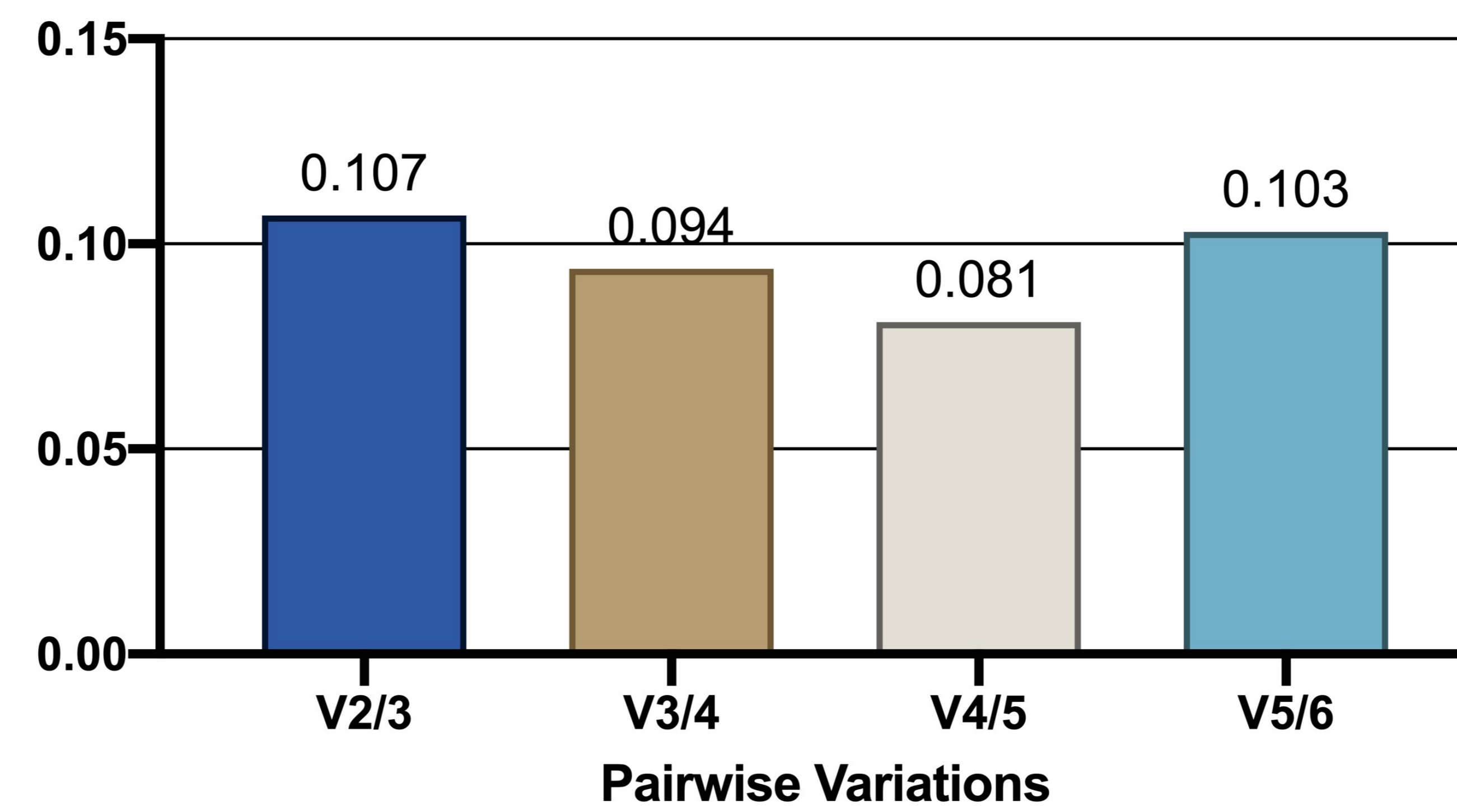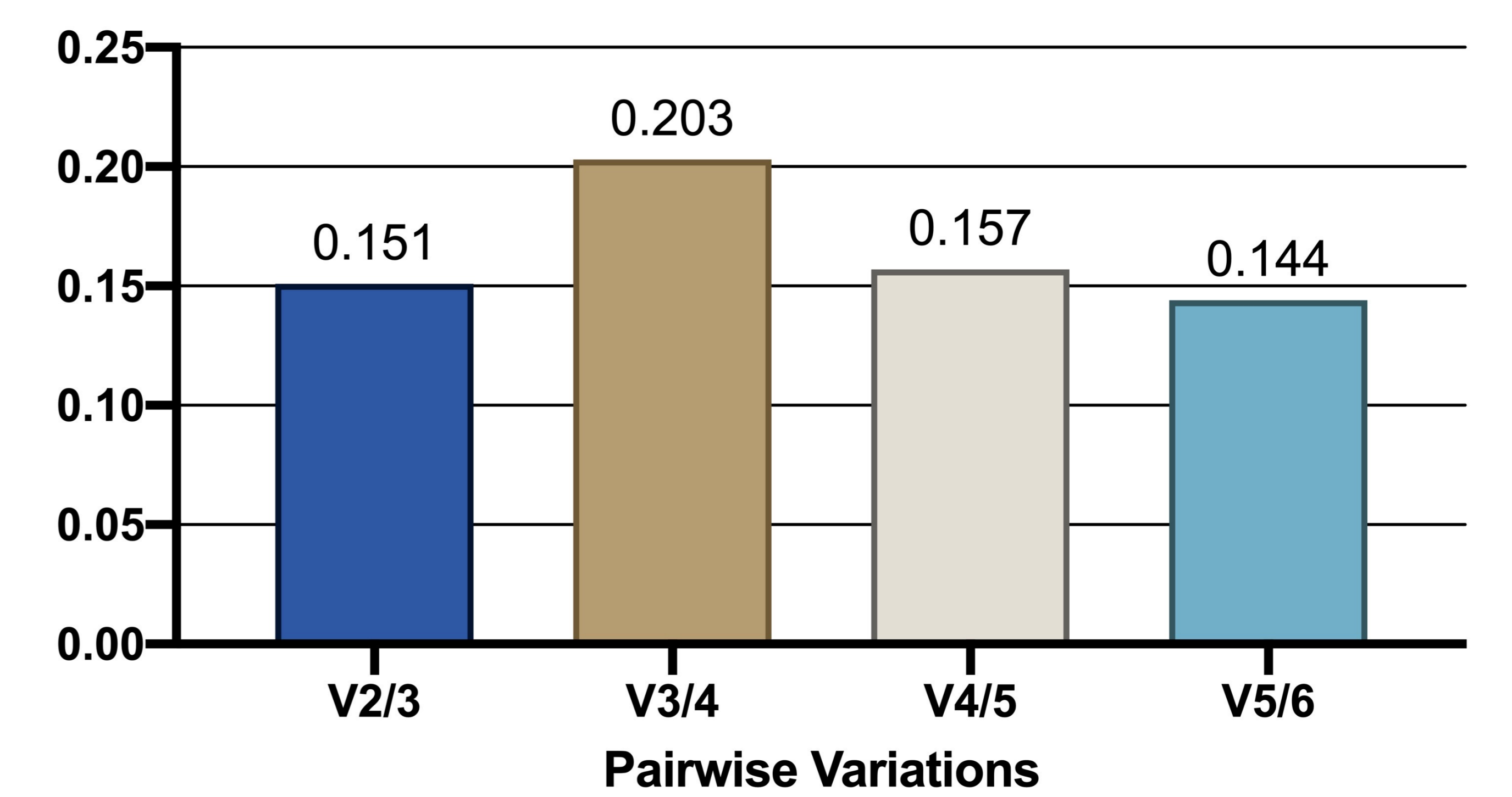

7 d

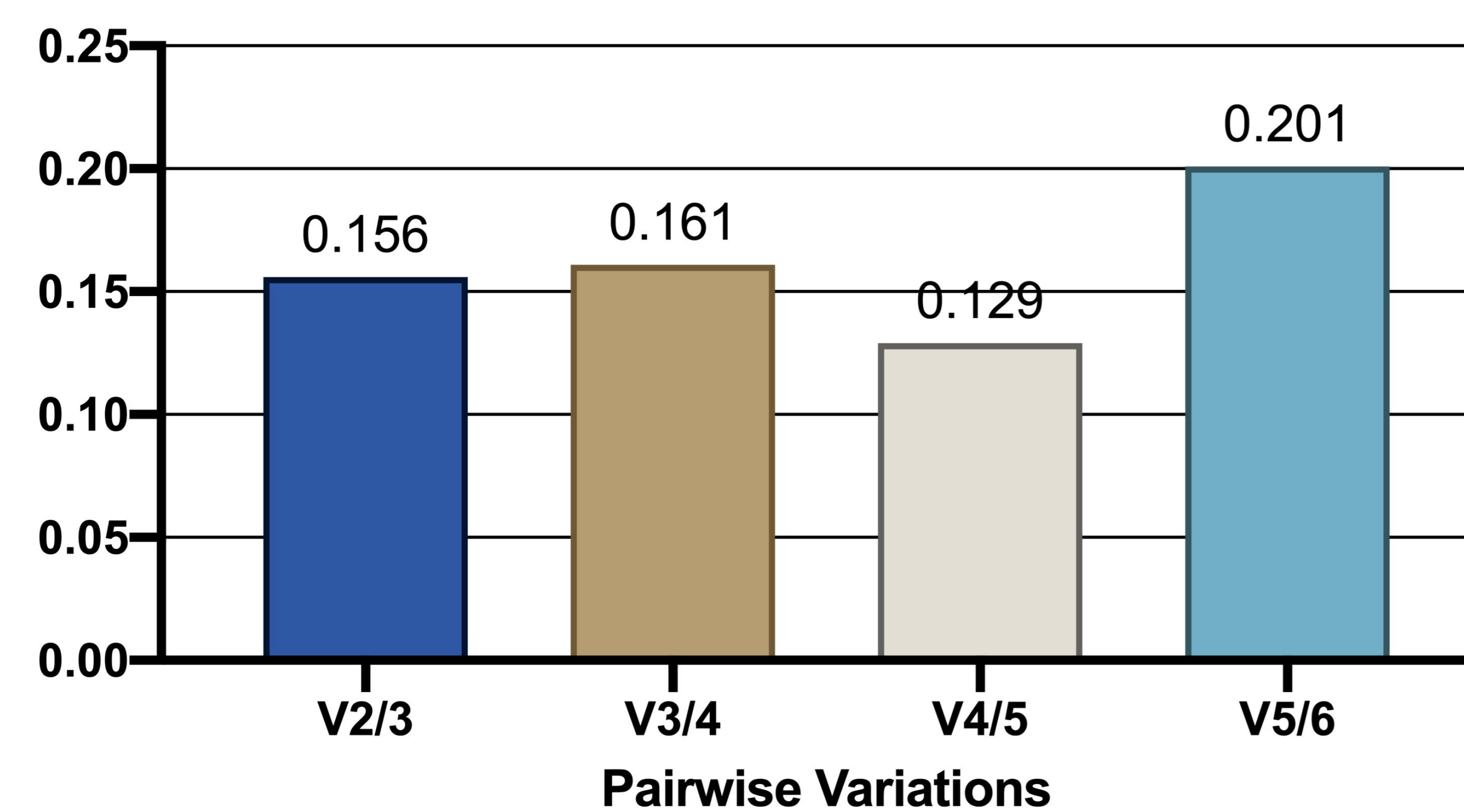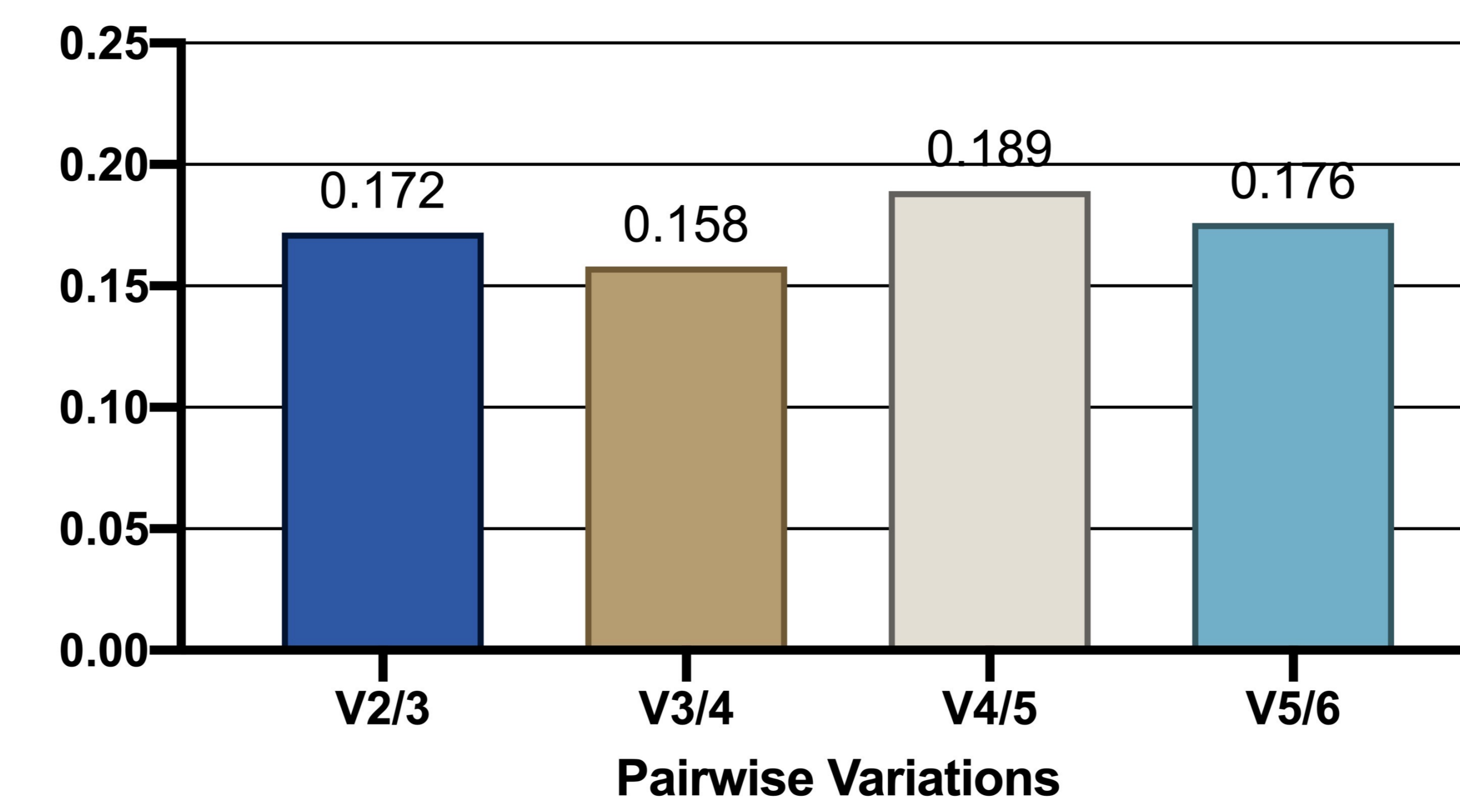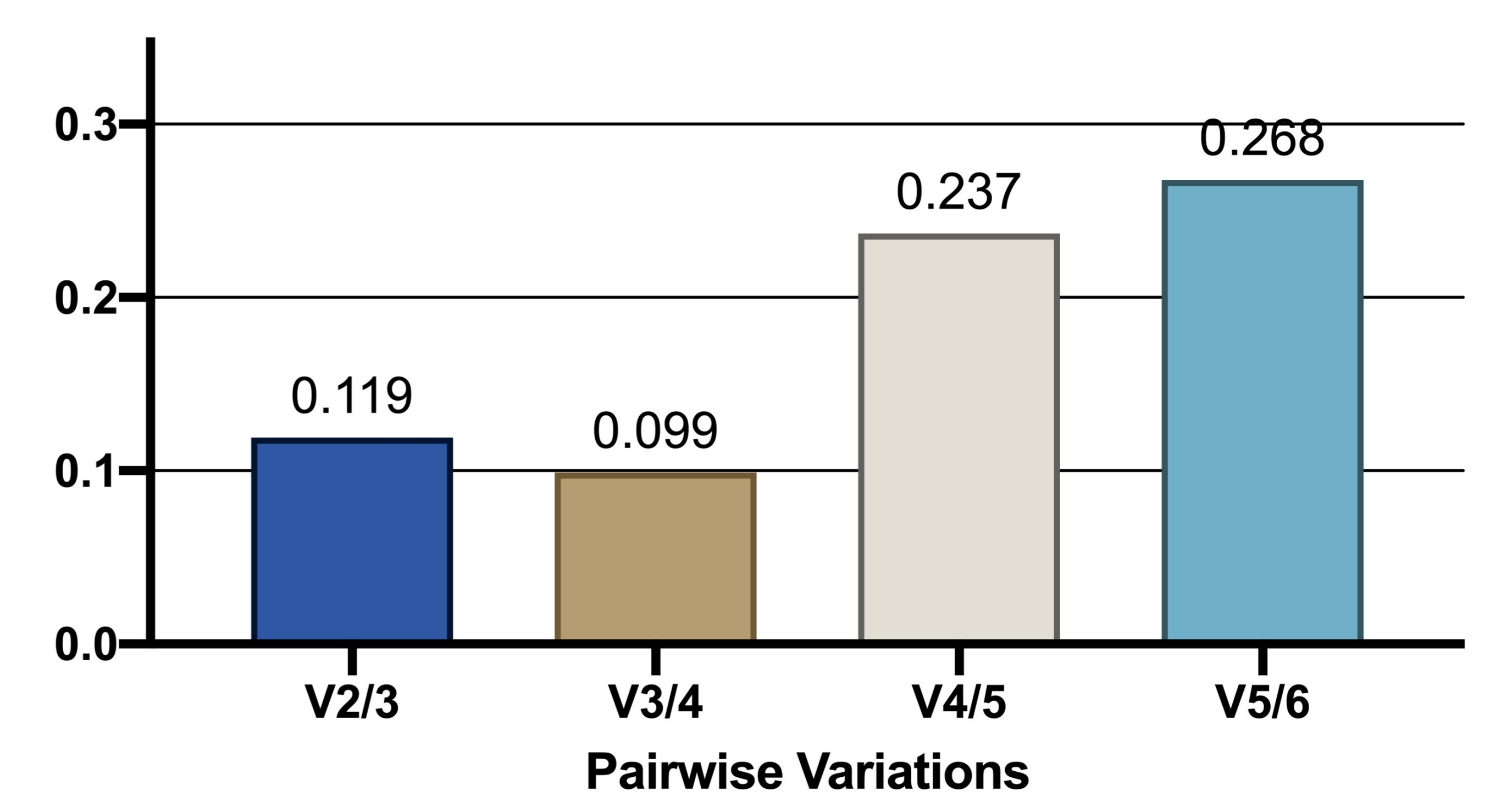

Supplement: Supplementary file 3 — Figure S3 [file JCMM-26-3060-s003.pdf]
