# Supplementary material for: Identification of optimal reference genes for gene expression studies in a focal cerebral ischaemia model—Spatiotemporal effects
Source: J Cell Mol Med. 2022 Apr 22;26(10):3060–7. doi: 10.1111/jcmm.17284 (PMC9097850; doi:10.1111/jcmm.17284)
Supplement: Supplementary file 4 — Supplementary Material [file JCMM-26-3060-s001.docx]

**SUPPLEMENTAL MATERIALS TO THE MANUSCRIPS:**

**Identification of optimal reference genes for gene expression studies in a focal cerebral ischemia model – spatiotemporal effects**

^1*^Pomierny Bartosz, ^1^Krzyzanowska Weronika, ^1^Jurczyk Jakub, ^2^Strach Beata, ^1^Skorkowska Alicja, ^1^Leonovich Innesa, ^1^Budziszewska Bogusława, ^2*^Pera Joanna

^1^Department of Toxicological Biochemistry, Faculty of Pharmacy, Jagiellonian University Medical College, Medyczna 9; 30-688 Kraków

^2^Department of Neurology, Faculty of Medicine, Jagiellonian University Medical College, Botaniczna 3; 31-503 Kraków

*Corresponding authors

Joanna Pera, [joanna.pera@uj.edu.pl](mailto:joanna.pera@uj.edu.pl); ORCID: 0000-0001-6142-2969

Bartosz Pomierny, [bartosz.pomierny@uj.edu.pl](mailto:bartosz.pomierny@uj.edu.pl); ORCID: 0000-0002-5352-5324

**Declarations**

*Funding.* The study was supported by National Science Center in Poland, grant no. 2016/21/B/NZ4/03294 and 2016/21/D/NZ4/03302. Beata Strach acknowledges the fellowship with the project POWR.03.02.00-00-I013/16.

*Consent for Publication.* All authors have given final approval of the version and agreed with the publication of this study here.

*Consent to Participate*. Not applicable.

*Conflict of Interest.* The authors declare that they have no conflict of interest.

*Ethics Approval.* All experimental protocols were approved by the First Local Ethical Committee at Jagiellonian University in Krakow (permit no: 11/2017 & 12/2017).

*Code availability.* Not applicable.

*Availability of data and material.* Not applicable.

*Authors` contributions.* BP, BB and JP contributed to the study design. BP, WK, JJ and BS contributed to the preparation of the animal model of focal cerebral ischemia. BP, WK, JJ, BS, AS, IL contributed to the carrying out of the tissue homogenization, reverse transcription and RT-PCR reaction. BP, WK, BB and JP contributed to the data analysis and manuscript preparation. All authors contributed to the data interpretation and approved the final version of the manuscript.

**Methods**

**Animals and experimental design.** All experiments were performed on male Sprague-Dawley rats (280-320 g, Charles Rivers). Animals were randomly allocated into the following groups: the SHAM 12 h, tMCAO 12 h, SHAM 24 h, tMCAO 24 h, SHAM 3 d, tMCAO 3 d, SHAM 7 d, and tMCAO 7 d groups. For each group, n = 8. Timepoint refers to the time lapse between the onset of reperfusion and animal decapitation and tissue collection. The animals were maintained on a normal day-night cycle at 22±2°C with free access to food and water. All experimental protocols were in accordance with the Guide for the Care and Use of Laboratory Animals published by the National Institutes of Health and were approved by the First Local Ethical Committee at Jagiellonian University in Krakow (permit no: 11/2017 & 12/2017). All studies involving animals are reported according to the ARRIVE (Animal Research: Reporting of In Vivo Experiments) guidelines, including the procedure for blinding the investigators to the identities of the animals at each point of the experiment.

**Focal cerebral ischemia model.** tMCAO was elicited according to the method of Longa et al. to induce transient focal cerebral ischemia as previously described (Krzyzanowska et al., 2016; Krzyżanowska et al., 2017). All surgical procedures were carried out under a stereoscopic microscope (Leica, A60F; Germany), and body temperature was maintained at a physiological level using a heating blanket (homeothermic blanket system; Harvard Apparatus). Arterial occlusion was confirmed using a blood flowmeter (PeriFlux System 6000; Perimed, Sweden), and a 70% blood flow reduction was considered to indicate a successful procedure. The rats were anesthetized with 5% isoflurane for induction and 2.5% isoflurane for maintenance. After exposure of the left external carotid artery (ECA), the internal carotid artery (ICA), and the common carotid artery (CCA), all branches of the ECA were coagulated, and the artery was ligated and cut. The ICA and CCA were temporarily secured with microvascular clips. A silicone-coated filament (Doccol, USA) was introduced into the lumen of the ECA and advanced until the blood flow decreased. The clip was removed from the CCA, and the wound was secured with silk sutures. The occlusion was maintained for 90 minutes. Afterwards, the wound was reopened, and the filament was removed to restore blood flow. The wound was closed with sutures. The sham operation was carried out as described above without insertion of the filament. Neurological deficits were assessed 12 h, 24 h, 3 d and 7 d after surgery. The 10-point grading system established by Philips et al., 2000 was used as described previously (Krzyzanowska et al., 2016; Phillips et al., 2000). Briefly, 0 points indicated no neurological deficit, whereas 10 points indicated the maximal neurological deficit. In a separate group of animals, the presence and size of brain infarction was assessed by 2,3,5-triphenyltetrazolium chloride (TTC) staining. Briefly, 2 mm thick coronal sections were stained with 1% TTC solution at 37°C for 10 min in the dark. Next, the brain sections were fixed with 10% paraformaldehyde for 30 min, and each section was photographed using a CCD camera (Motic) (Fig.S1). Coronal sections were collected from region, relative to bregma: +5.2 mm to -6.2 mm.

**RT-qPCR.** The rats were decapitated 12 h, 24 h, 3 d, or 7 d after reperfusion or sham operation. The brains were removed, and the selected ipsilateral brain structures (CX, HIP, and DS) were isolated and immersed in *fixRNA* solution (EURx, Poland) for 24 h at 4°C to preserve RNA. Next, total RNA was extracted using TRIzol reagent and purified with a microcolumn system according to the manufacturer’s protocol. The concentration and purity of the RNA were determined by measuring the A260/A280 ratio with a Nanoquant plate (Tecan, Austria). cDNA was synthesized using 2 µg of total RNA and a Smart First Strand cDNA Synthesis Kit (EURx, Poland) according to the manufacturer’s protocol. The cDNA was stored at -80°C until use. RT-qPCR amplification was performed on a CFX Connect system (Bio-Rad, USA) using appropriate TaqMan Gene Expression Assays (Applied Biosystems, USA), 200 ng of template cDNA and Probe qPCR Master Mix (EURx, Poland) according to the manufacturer’s instructions. The thermal cycling conditions were as follows: 95°C for 15 min (initial denaturation) followed by 35 cycles of 94°C for 15 s (denaturation), 55°C for 30 s (annealing) and 72°C for 30 s (extension). Reactions for each sample were performed in triplicate. The efficiency of the PCR for each TaqMan probe was verified. The fold change in each gene expression was calculated using the ΔCt method. Next, these values or Ct values were used for further statistical analysis. The TaqMan probes used for RT-qPCR are listed in Table 1.

**Statistical Analyses.** Ct values for each gene, brain structure and timepoint are expressed as the mean ± standard error of the mean (SEM). To determine changes in the expression of a particular gene in a selected brain structure and at a specific timepoint, one-way ANOVA followed by Dunnett’s test was performed. For each comparison, the tMCAO group was compared to the sham group at the same timepoint. A calculated *p* value < 0.05 was considered statistically significant. Calculations were performed using GraphPad Prism software ver. 8.2.1 (GraphPad Software, USA). To compare the stability of the investigated genes between the ischemic and sham groups at a particular timepoint, one-way ANOVA was used. For this analysis, the Ct value of a particular gene in tMCAO rats was linearized by transformation with the 2^-ΔCt^ method and compared to the value in sham animals at the same timepoint. Then, variance analysis was performed to compare the control group (sham group at four timepoints) with animals subjected to tMCAO sacrificed 12 h, 24 h 3 d, or 7 d after reperfusion.

To determine the stability of RG candidates, four statistical methods were used: GeNorm, NormFinder, BestKeeper, ΔCt. Next, ranked lists were integrated using the geometrical mean method. For GeNorm and NormFinder analysis, relative nonnormalized quantities were used as input data for analysis, whereas for BestKeeper analysis, Ct values were used. GeNorm analysis was performed using qbase+ software ver. 3.2 (Biogazelle, Belgium). NormFinder and BestKeeper analysis were performed using publicly available Excel sheet-based software. The ΔCt method was performed using CFX Maestro Software (Bio-Rad, USA).

**References**

Krzyzanowska, W., Pomierny, B., Budziszewska, B., Filip, M., & Pera, J. (2016). N-Acetylcysteine and Ceftriaxone as Preconditioning Strategies in Focal Brain Ischemia: Influence on Glutamate Transporters Expression. *Neurotoxicity Research*, *29*(4), 539–550. https://doi.org/10.1007/s12640-016-9602-z

Krzyżanowska, W., Pomierny, B., Bystrowska, B., Pomierny-Chamioło, L., Filip, M., Budziszewska, B., & Pera, J. (2017). Ceftriaxone- and N-acetylcysteine-induced brain tolerance to ischemia: Influence on glutamate levels in focal cerebral ischemia. *PLoS ONE*, *12*(10), e0186243. https://doi.org/10.1371/journal.pone.0186243

Phillips, J. B., Williams, A. J., Adams, J., Elliott, P. J., & Tortella, F. C. (2000). Proteasome inhibitor PS519 reduces infarction and attenuates leukocyte infiltration in a rat model of focal cerebral ischemia. *Stroke*, *31*(7), 1686–1693. https://doi.org/10.1161/01.STR.31.7.1686
